# The Therapeutic Effectiveness of the Coadministration of Weekly Risedronate and Proton Pump Inhibitor in Osteoporosis Treatment

**DOI:** 10.1155/2014/607145

**Published:** 2014-11-09

**Authors:** Mizue Tanaka, Soichiro Itoh, Taro Yoshioka, Kimihiro Yamashita

**Affiliations:** ^1^Department of Orthopaedic Surgery, Kawakita General Hospital, 1-7-3 Asagaya-kita, Suginami-ku, Tokyo 166-8588, Japan; ^2^Department of Orthopaedic Surgery, Sakurakai Hospital, 2-13-1 Senju Sakuragi, Adachi-ku, Tokyo 120-0045, Japan; ^3^Department of Inorganic Materials, Institute of Biomaterials and Bioengineering, Tokyo Medical and Dental University, 2-3-10 Kanda-Surugadai, Chiyoda, Tokyo 101-0062, Japan

## Abstract

This trial was conducted to investigate the long-term effects of proton pump inhibitor (PPI) coadministration on the efficacy of weekly risedronate treatment for osteoporosis. Ninety-six women over 50 years old with low bone mineral density (BMD) participated in this trial. Subjects were randomly divided into 2 groups: a 17.5 mg dose of sodium risedronate was administered weekly, with or without a daily 10 mg dose of sodium rabeprazole (*n* = 49 and 47 in the BP + PPI and BP groups, resp.). The following biomarkers were measured at the baseline and every 3 months: bone-specific alkaline phosphatase, N-terminal telopeptide of type I collagen corrected for creatinine, parathyroid hormone, BMD of the lumbar spine, and physical parameters evaluated according to the SF-36v2 Health Survey. Statistical comparisons of these parameters were performed after 6, 12, 18, and 24 months. The Δ values of improvement in physical functioning after 12 months and bodily pain after 6 and 12 months in the BP + PPI group were significantly larger than those in the BP group. These results suggest that PPI does not adversely affect bone metabolism. Alternatively, approved bone formation by concomitant PPI treatment may have had favorable effects on the improvement of bodily pain and physical functions.

## 1. Introduction

Various types of therapeutic products for osteoporosis have been developed. Among them, bisphosphonate (BP) is currently the most commonly prescribed drug for the treatment of osteoporosis. This compound can selectively inhibit osteoclast-mediated bone resorption, thereby resulting in an increase in bone mineral density (BMD). Pyridinyl BP risedronate ([1-hydroxy-2-(3-pyridinyl)-ethylidene] bis[phosphonic acid] monosodium salt) in the risedronate group is effective for the treatment of postmenopausal osteoporosis and reduces the risk of vertebral fracture within the first year of treatment [1, 2]. Gastrointestinal medicine is occasionally used to prevent upper gastrointestinal (GI) tract adverse events for the prolonged administration of risedronate. It is reported that the infusion of ranitidine, which is a histamine H2-receptor antagonist that inhibits stomach acid production, increases the gastric pH and doubles the bioavailability of 4-amino-1-hydroxybutylidene-1,1-bisphosphonate monosodium [3]. Hence, it is expected that risedronate administered in combination with a proton pump inhibitor (PPI) may be more effective than the administration of risedronate alone in inhibiting bone resorption and treating osteoporosis as well as preventing GI tract adverse events. Therefore, Itoh et al. conducted a prospective, randomized trial to study the effects of sodium risedronate hydrate used adjunctively with sodium rabeprazole on the dynamic state of bone metabolism [4]. The increase in BMD and the improvement of physical functioning in the group in which risedronate was coadministered with rabeprazole were significantly larger, while the decrease in bone-specific alkaline phosphatase (BAP) in the coadministration group was significantly smaller than that in the group in which only risedronate was administered. This fact demonstrates that rabeprazole does not adversely affect bone metabolism without inducing secondary hyperparathyroidism, most likely by facilitating the effect of risedronate by increasing its bioavailability, as long as rabeprazole is administered in a therapeutic dose and not for an extended period. The authors conclude that risedronate administration in combination with a PPI may be more effective than treatment with risedronate alone, not only for treating osteoporosis but also for improving physical fitness. In this trial, we investigated the effects of the coadministration of a PPI on the efficacy of weekly risedronate treatment for osteoporosis during long-term use.

## 2. Materials and Methods

We screened Japanese female patients over 50 years of age with low bone mass who visited our hospital for a medical checkup or for osteoporosis treatment between June 2009 and December 2013. The BMD of trabecular bone was measured at the 3rd lumbar vertebra (L3) with a quantitative computer tomography (Aquilion TSX-101A, Toshiba Medical Systems Co., Tokyo, Japan). The BMD values were estimated from the CT number using a bone mineral reference phantom (Kyoto Kagaku Co., Ltd, Kyoto, Japan) [5]. The young adult mean (YAM) provided from the manufacturer was 181.4 ± 13.1 mg/cm^3^ (average ± standard deviation: SD). We classified a patient as having low bone mass when her BMD was 2.5 SD below the YAM provided by the manufacturer and enrolled her in this study. When a wedge deformity of the L3 was found, an alternative vertebral body of the upper or lower lumbar spine without a deformity was instead used for the measurement. Patients who had taken osteoprotective medications or PPIs previously were excluded from this study. Patients who had a preexisting medical illness, were taking medications known to affect BMD, or could not continue self-administration were also excluded. The trial was approved by the ethics committees of the authors' institutions and was conducted according to the Declaration of Helsinki. Informed consent was obtained from each candidate. The 96 women who participated in this trial were randomly divided into 2 groups in which a 17.5 mg dose of sodium risedronate was administered weekly with or without a daily 10 mg dose of sodium rabeprazole for 24 months consecutively. The number of patients who were administered risedronate with or without rabeprazole (BP + PPI and BP group, resp.) was 49 and 47, respectively. Patients were permitted to continue their use of other medications with the exception of other osteoporosis treatments and PPIs, as well as nonsteroidal anti-inflammatory drugs (NSAIDs). Supplementary calcium of 300 mg daily was administered orally from the registration date until the end of the study. Concomitant use of any drug considered to affect bone metabolism, including vitamin D, was prohibited during the study. All patients received rehabilitation centered around general muscle strengthening, depending on their exercise capacities.

The following biomarkers were measured at the baseline and every 3 months in addition to the routine blood screening: serous BAP, N-terminal telopeptide of type I collagen (NTX) in the urine, parathyroid hormone (PTH), phosphate, calcium, and the BMD of L3. The NTX value was corrected for creatinine levels and expressed as NTX/Cr. Improvements in physical fitness, physical functioning, and bodily pain were evaluated by the SF-36v2 Health Survey (United Health Group Inc., Minneapolis, MN). Adverse events information was collected every month during the trial.

The number of patients in the trial who experienced adverse events and fracture was statistically compared between the groups using a Chi-squared for independence test. Differences were statistically significant when *P* < 0.05. The differences of the background data consisting of age, body weight and height, years since menopause, renal function indicated by creatinine and serum levels of calcium and phosphate, and the values of the BMD, BAP, NTX/Cr, PTH, and the physical parameters in the BP and BP + PPI groups were statistically compared to match the parameters in each group at baseline. The population variance between these groups was compared using a Mann-Whitney *U* test.

The absolute values of the differences between the values and at the baseline (Δ value) were determined in BMD, BAP, NTX/Cr, PTH, and physical parameters to compare the improvement in low bone mass imparted by this treatment. Statistical significance was evaluated for the Δ value of these parameters between the BP group and BP + PPI group using a Mann-Whitney *U* test (*P* < 0.05).

## 3. Results

In the BP and BP + PPI groups, 10 and 9 adverse events were reported, respectively, as summarized in Table 1. There was one case each of ileus and leukopenia in the BP + PPI group, and these patients were eliminated from the analysis. As a result, the number of patients who were enrolled in this trial was 47 in each group. Furthermore, the PTH serum concentration rose in 2 cases, suggesting secondary hyperparathyroidism in the BP + PPI group. Wedge deformity of vertebrae advanced without trauma in 1 and 2 cases in the BP and BP + PPI groups, respectively. There were no significant differences between groups in the number of onset of adverse events and occurrence of fracture. The background data consisting of age, body weight and height, years since menopause, renal function, and the values of the BMD, BAP, NTX/Cr, PTH, and the physical parameters at the baseline are summarized in Table 2. Because there were no significant differences between groups in the values, it was confirmed that the patients in each group were matched for the parameters at baseline.

The Δ values of BMD, BAP, NTX/Cr, PTH, and the physical parameters are summarized in Table 3. Although there were no significant differences between the groups in these Δ values, there was a tendency that the increase of BMD and decrease of NTX/Cr were larger and decrease of BAP was smaller in the BP + PPI group than in the BP group. The Δ values of improvement in physical functioning after 12 months and bodily pain after 6 and 12 months in the BP + PPI group were significantly larger than those in the BP group. There was no significant difference between the groups in the increase of PTH.

## 4. Discussion

PPIs are reported to help the effective absorption of BP, which should increase the bioavailability of BP in vivo and decrease bone resorption [3]. In contrast, it is also known that PPIs reduce calcium absorption by raising the gastric pH [6, 7] and, subsequently, may increase PTH secretion, which would lead to an increase in bone resorption. Indeed, we observed an increase in the PTH serum concentration in 2 cases, which rose presumed hyperparathyroidism in the BP + PPI group. Thus, a PPI may affect bone metabolism negatively by affecting calcium metabolism or positively in combination with BP. Therefore, it is unclear whether these conflicting effects would lead to improved or worsened bone metabolism. In a previous study of Itoh et al., the authors investigated the short-term effects of the coadministration of a PPI on the efficacy of risedronate treatment for osteoporosis up to 9 months after administration [4]. The increase in BMD and improvement of physical functioning in the BP + PPI group were significantly larger, and the decrease in BAP in the BP + PPI group was significantly smaller than that of the BP group. They concluded that risedronate administration in combination with a PPI may be more effective not only for treating osteoporosis but also for improving physical fitness than treatment with risedronate alone. We designed a study on the coadministration of a PPI and once-a-week risedronate and examined the long-term effects of this regimen on postmenopausal osteoporosis treatment. As a result, although there was no significant difference between the groups, BMD tended to increase more, the NTX/Cr value decrease more, and the BAP value decrease less in the BP + PPI group compared with those in the BP group through the administration period. Furthermore, at an early stage, physical functioning and bodily pain improved significantly with risedronate administration combined with PPI. These results demonstrate that PPI does not adversely affect bone metabolism but facilitates the effect of risedronate most likely by increasing its absorption in the small intestine as previously reported [3], which should increase the bioavailability of bisphosphonate in vivo. Thus, approved bone formation by risedronate with concomitant PPI treatment improved bodily pain after 6 and 12 months, followed by physical function after 12 months in the BP + PPI group. Increased level of bone absorption mediated by osteoclasts weakens the bone structural integrity and results in painful and potentially debilitating skeletal-related events including vertebral and nonvertebral fracture. Pain and these evil events undermine quality of life (QOL) and compromise functional independence of patients. Previous clinical trial assessments, however, have focused on frequencies of skeletal-related events mainly, instead of pain and physical functions. Our results suggest that evaluation of these physical factors might be used as a surrogate for decreased QOL in patients with osteoporosis.

The dosages of BPs used in Japan are half the dosages used outside Japan as in this trial: alendronate 5 mg daily or 35 mg weekly and risedronate 2.5 mg daily or 17.5 mg weekly [8–11]. The phase III, multicenter, randomized, and double-blind trial was conducted for 2 years to compare the effects on involutional osteoporosis and tolerability of 75 mg once-a-month oral risedronate with those of 2.5 mg daily regimen in Japanese patients [12]. As a result, 75 mg once-a-month oral risedronate had equivalent efficacy in involutional osteoporosis therapy and was similarly well tolerated compared with the daily 2.5 mg regimen in Japanese patients. It is concluded that the sufficient dose of once-a-month risedronate for involutional osteoporosis treatment is 75 mg, which is half that used outside Japan (150 mg) consistent with the once-daily and once-weekly dosage. Another study examined the tolerability and pharmacokinetics of risedronate after a single oral administration and during multiple oral administrations in healthy Japanese adult male volunteers [13]. The results showed that risedronate was well tolerated when delivered as a single administration of up to 20 mg or as a multiple administration of up to 5 mg/day. The pharmacokinetic profile of risedronate was considered to show linearity in a dosage range of up to 20 mg. The plasma concentrations of risedronate after the administration of 2.5 mg risedronate to the Japanese population were nearly comparable to the serum concentrations after the administration of 5 mg risedronate to the United Kingdom study population. These facts suggest that there is a racial difference in bioavailability of BPs; however, the reason for this difference remains unknown. Therefore, further detailed investigations including non-Japanese patients will be anticipated to conclude the efficacy of BP administration with a PPI on osteoporosis treatments, containing that on fragile fractures because recent evidence suggests that PPI use for an extended period may affect fracture risk and, moreover, that concomitant use of a PPI may wane the antifracture effect of BP [14, 15].

This trial has several limitations. First, the present study was conducted in a single center, and the number of patients enrolled was relatively small, though there is an advantage in that patients of a homogeneous life environment were assembled. Second, because 25(OH)D was not measured, there remains a possibility that some cases of osteomalacia could have been included in this study. Therefore, the variation in the measured values of BAP and PTH cannot simply indicate the effects of BP administration in combination with PPI. Measurement of 25(OH)D is not currently approved by Japanese insurance, which hampered our efforts to address this point.

## 5. Conclusion

It is suggested that a PPI does not adversely affect bone metabolism, most likely due to the facilitating effect of risedronate through increasing its bioavailability. BP administration in combination with a PPI may be more effective than treatment with risedronate alone, not only for treating osteoporosis but also for improving bodily pain and physical functions, as long as the PPI is administered in a therapeutic dose and not for an extended period.

## Figures and Tables

**Table 1:** Adverse events occurred under medical treatment.

BP	*N*	BP + PPI	*N*
Stomachache	2	Stomachache	1
Constipation	1	Heavy stomach feeling	1
Gingival bleeding	1	Constipation	1
Drowsiness	1	Double vision	1
Reflux esophagitis	1	Ileus	1
Neck discomfort	1	Leukopenia	1
Gaseous regurgitation	1	Vertebral fracture	2
Urticaria	1	Nausea	1
Vertebral fracture	1		

Total number of adverse events	10		9

**Table 2:** The background data, including age, body weight, body height, years since menopause, serum levels of calcium, phosphate and renal function indicated by creatinine, and the measured numeric values of the BMD, NTX/Cr, BAP, PTH, and the physical parameters at the baseline.

Baseline	BP	BP + PPI
Number of cases	47	47
Age (years old)	70.1 ± 8.5	68.8 ± 8.5
Body weight (Kg)	50.1 ± 7.8	51.9 ± 7.1
Body height (cm)	150.1 ± 9.5	151.8 ± 8.6
Years since menopause	20.0 ± 14.3	18.2 ± 11.2
Calcium (mg/dL)	9.2 ± 0.5	9.2 ± 0.6
Phosphate (mg/dL)	3.7 ± 0.3	3.6 ± 0.5
Creatinine (mg/dL)	0.6 ± 0.1	0.8 ± 0.8
BMD (mg/cm^3^) YAM 181.4 ± 13.1	66.3 ± 36.8	55.5 ± 31.1
NTX/Cr (nmolBCE/mmol*·*Cr)	55.8 ± 16.1	63.4 ± 19.3
BAP (U/L)	17.3 ± 6.6	20.7 ± 13.2
PTH (pg/mL)	41.0 ± 10.5	43.0 ± 16.2
Physical functioning	40.4 ± 15.2	33.5 ± 14.5
Bodily pain	41.2 ± 9.4	36.5 ± 9.5

Average ± standard deviation.

**Table 3:** The Δ value in BMD, NTX/Cr, BAP, PTH, and the physical parameters.

	BP	BP + PPI
Increase of BMD (mg/cm^3^)		
6 M	2.9 ± 11.5	7.1 ± 7.5
12 M	6.0 ± 23.2	8.0 ± 12.4
18 M	13.3 ± 9.2	13.0 ± 10.6
24 M	15.2 ± 11.2	17.5 ± 3.6
Decrease of NTX/Cr (nmolBCE/mmol*·*Cr)		
6 M	24.1 ± 23.0	27.4 ± 18.3
12 M	29.8 ± 20.4	33.1 ± 22.2
18 M	28.5 ± 20.1	34.4 ± 20.0
24 M	40.2 ± 10.2	45.6 ± 20.7
Decrease of BAP (U/L)		
6 M	4.9 ± 3.8	4.7 ± 5.7
12 M	6.3 ± 3.5	4.6 ± 7.0
18 M	6.2 ± 4.3	5.8 ± 7.2
24 M	7.0 ± 1.2	6.1 ± 5.6
Increase of PTH (pg/mL)		
6 M	0.9 ± 17.5	2.6 ± 12.1
12 M	3.2 ± 11.1	2.7 ± 10.0
18 M	3.6 ± 6.4	4.3 ± 6.9
24 M	5.3 ± 10.0	5.0 ± 12.4
Improvement of physical functioning		
6 M	4.7 ± 6.4	8.7 ± 7.1
12 M	4.0 ± 7.7	11.2 ± 9.4^*^
18 M	4.5 ± 7.5	9.9 ± 7.3
24 M	9.4 ± 14.2	10.0 ± 6.5
Improvement of bodily pain		
6 M	4.1 ± 10.0	11.9 ± 3.6^*^
12 M	4.5 ± 11.1	12.2 ± 12.3^**^
18 M	11.6 ± 8.7	12.3 ± 10.3
24 M	13.6 ± 7.0	14.5 ± 9.3

Average ± standard deviation. ^*^ *P* < 0.05, ^**^ *P* < 0.01.
